# Photo Quiz: A 51-year-old man with a lung mass—a multidisciplinary diagnosis

**DOI:** 10.1128/jcm.01557-23

**Published:** 2024-07-16

**Authors:** Anthony R. Russo, Maxwell T. Roth, Eric P. Grewal, Eric S. Rosenberg, John A. Branda

**Affiliations:** 1 Department of Pathology, Massachusetts General Hospital, Boston, Massachusetts, USA; 2 Department of Medicine, Massachusetts General Hospital, Boston, Massachusetts, USA; Mayo Clinic Minnesota, Rochester, Minnesota, USA

## PHOTO QUIZ 

**Fig 1:**
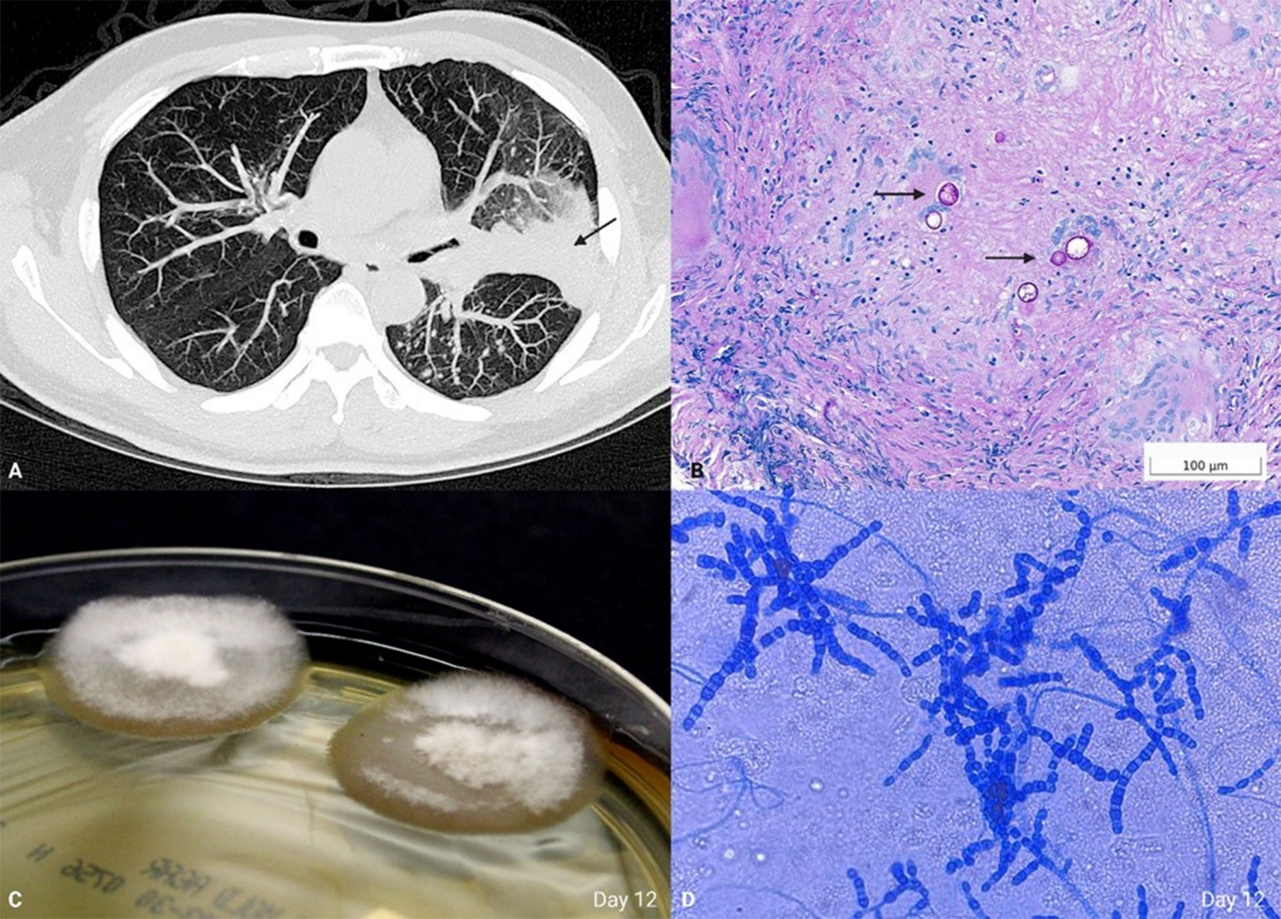
(**A**) Non-contrast chest computed tomography (CT), lung window, axial. Consolidative opacity (arrow). (**B**) Permanent tissue histology of the transbronchial biopsy with periodic acid-Schiff with diastase (PASD)-positive spherules (arrows), 400×. (**C**) Sabouraud dextrose agar, day 12. (**D**) Lactophenol cotton blue preparation, 200×.

A 51-year-old male, never smoker, presented to the pulmonary clinic with 6 weeks of dry cough, dyspnea, myalgias, and intermittent low-grade fevers. The patient resided in suburban New England, and history was notable for frequent travel to China, Arizona, and Florida. A course of azithromycin failed to improve the patient’s symptoms, prompting a chest x-ray which noted a left-sided air-space opacity. A follow-up non-contrast chest CT (Fig. 1A) revealed a posterior left-upper-lobe consolidative opacity with associated hilar and mediastinal lymphadenopathy. The radiographic findings were most compatible with malignancy, but the differential diagnosis included infectious and autoimmune etiologies. Serum 1-3 β-D-Glucan and bronchoalveolar lavage fluid Aspergillus galactomannan antigens were negative. Nasopharyngeal swab PCR for SARS-CoV-2 and T Spot TB test were negative.

Given the concerning imaging findings and differential diagnosis, a decision was made to proceed with endobronchial ultrasound and tissue biopsy. Transbronchial biopsy, evaluated intraoperatively by frozen section, revealed granulomatous inflammation, prompting tissue to be sent to the microbiology laboratory for bacterial, mycobacterial, and fungal culture. A slow-growing tan-waxy mold with fine white aerial hyphae produced mature colonies in fungal culture on day 12 using routine culture media (Fig. 1C). Lactophenol cotton blue preparation revealed alternating barrel-shaped arthroconidia in a background of thin septated-hyphae (Fig. 1D). Bacterial and mycobacterial cultures were negative. What is your diagnosis?

## ANSWER TO PHOTO QUIZ

The pathogen was identified as presumptive *Coccidioides* by identification of the characteristic alternating barrel-shaped arthroconidia in a background of thin septated-hyphae on a lactophenol cotton blue preparation (Fig. 1D). Culture confirmation was performed by genetic sequencing at an outside reference laboratory. A diagnosis of Coccidioidomycosis was confirmed. Alternatively, culture confirmation can be performed using MALDI-TOF MS, but due to significant biosafety concerns with performing diagnostic procedures outside a biosafety cabinet, this was not done in our laboratory.

Permanent tissue histology demonstrated granulomatous inflammation with necrosis and rare-scattered degenerate fractured spherules, highlighted by PAS-D, consistent with coccidioides (Fig. 1B). The spherules are immature as they do not contain endospores and, upon higher power examination, were shown to be non-budding.

Prior to biopsy, serological testing for coccidioides-specific-IgM was negative, along with a positive coccidioides IgG and complement fixation (1:2). Initially, the negative IgM result along with a low titer IgG was of limited value because it could have represented a prior exposure and not be indicative of active disease; thus, a decision was made to proceed with direct tissue sampling for definitive diagnosis. In hindsight, the negative IgM and low titer IgG are likely the result of a prolonged course between initial exposure and presentation. Additional serologic testing for 1-3 β-D-glucan revealed a negative result (<60 pg/mL); however, BDG is variably elevated in acute Coccidioides infection and is not a reliable indicator of active disease (1). BAL Aspergillus galactomannan antigen testing was negative, which is the expected result in cases of coccidioidomycosis.

This patient’s case of coccidioidomycosis was attributed to remote exposure while traveling to Arizona. Also known as valley fever, *Coccidioides* is encountered most frequently as an environmental organism from soil in the southwestern United States, Mexico, and Central America (2). Most coccidioides infections are self-limited, although disseminated disease can occur in a small fraction of patients. Occasionally, patients develop persistent disease characterized by pulmonary nodules and coccidioidal cavity formation which can be difficult to distinguish from malignancy. In such cases, biopsy, histologic examination, and microbiologic studies are warranted. The patient was started on a course of fluconazole and, at a 4-week follow-up visit, reported marked improvement of his symptoms.

## References

[1] Thompson GR, Bays DJ, Johnson SM, Cohen SH, Pappagianis D, Finkelman MA. 2012. Serum (1->3)-β-D-glucan measurement in coccidioidomycosis. J Clin Microbiol 50:3060–3062. doi:10.1128/JCM.00631-12 22692738 PMC3421794

[2] Mazi PB, Sahrmann JM, Olsen MA, Coler-Reilly A, Rauseo AM, Pullen M, Zuniga-Moya JC, Powderly WG, Spec A. 2023. The geographic distribution of dimorphic mycoses in the United States for the modern era. Clin Infect Dis 76:1295–1301. doi:10.1093/cid/ciac882 36366776 PMC10319749

